# Genome assembly of *Pseudomonas* sp. strain SED1^T^, a psychrotolerant bacterium isolated from Deception Glacier (Washington, USA)

**DOI:** 10.1128/mra.00125-24

**Published:** 2024-03-25

**Authors:** Daniel H. Shain, Eric A. Klein

**Affiliations:** 1 Biology Department, Rutgers, The State University of New Jersey, Camden, New Jersey, USA; 2 Center for Computational and Integrative Biology, Rutgers, The State University of New Jersey, Camden, New Jersey, USA; Montana State University, Bozeman, Montana, USA

**Keywords:** psychrotolerant, *Pseudomonas*, glacier

## Abstract

Strain SED1^T^ was isolated from glacial samples collected on Mount Deception, Washington, USA. Genome sequencing and assembly identified a DNA G + C content of 60.4 mol% with 6,125 predicted proteins. Analysis by the Type Strain Genome Server is consistent with the isolate representing a previously undescribed species in the genus *Pseudomonas*.

## ANNOUNCEMENT

Samples from Mount Deception, Olympic National Park, Washington, USA (47°48′47″N 123°14′01″W) were collected in September 2016 using a small spade to transfer surface snow/ice into plastic containers. Samples were melted and grown on Hutner-imidazole-glucose-glutamate minimal media agar plates at 4°C to isolate psychrotolerant oligotrophic bacteria (1). Three distinct colony morphologies were observed; 16S rRNA sequencing identified one Janthinobacterium and two Pseudomonads, one of which (SED1^T^) is further characterized in this study. The 16S rRNA sequence was PCR-amplified using Q5 polymerase (New England Biolabs) and primers 27F (AGAGTTTGATCMTGGCTCAG) and 1492R (GGTTACCTTGTTACGACTT), followed by Sanger sequencing. BLAST identified the closest sequence match as *Pseudomonas yamanorum* (GenBank accession no: CP012400). Species of the genus *Pseudomonas* are rod-shaped, motile, aerobic, Gram-negative gammaproteobacteria. Pseudomonads inhabit a variety of environmental niches, including soil, water, and plant and animal hosts. A number of Pseudomonads are psychrotolerant (2–6), and these organisms provide an excellent model system for studying the mechanisms of cold tolerance.

**Fig 1:**
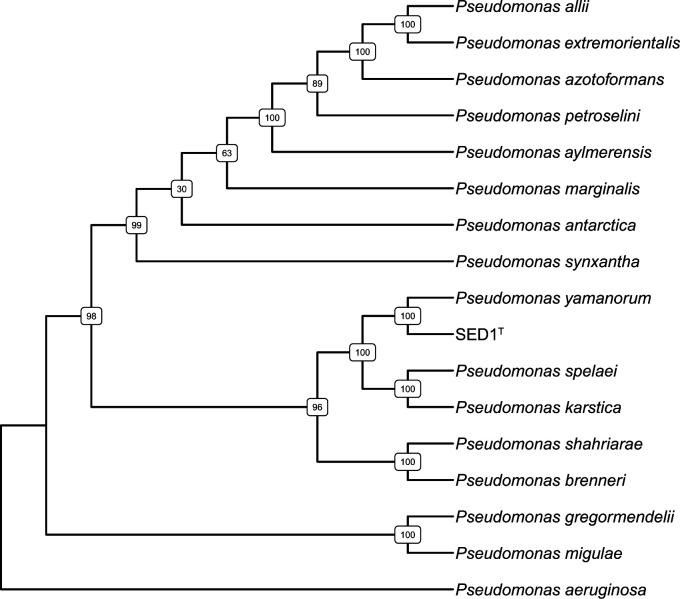
Multi-locus maximum-likelihood phylogenetic analysis of SED1^T^. The concatenated sequences of 16S rRNA, *dnaK*, *gyrB*, *recA*, *rpoD*, and *trpB* (8) from the indicated species of the *P. gessardii* subgroup were aligned using MUSCLE aligner (9), and phylogenetic trees were prepared using RAxML (version 8.2.12) with 100 bootstraps (10). Bootstrap percentages are indicated at the nodes.

Bacteria were grown in tryptic soy broth overnight at 22°C, genomic DNA was extracted (Qiagen Blood and Tissue Kit), and samples were sent to SeqCenter (Pittsburgh, PA, USA) for library preparation and sequencing. The DNA library was prepared using the Illumina DNA Prep kit and IDT 10 bp UDI indices. Sequencing was performed using the Illumina NextSeq2000 platform to produce 2 × 151-bp reads. Demultiplexing, quality control, and adapter trimming of the reads were performed with bcl-convert (v3.9.3) (Illumina). The total number of read pairs was 2,965,170 with a *Q*30 value of 92.27%. The reads were assembled using the NCBI RAPT pipeline (https://www.ncbi.nlm.nih.gov/rapt) using default settings. Gene annotation was performed using the NCBI Prokaryotic Genome Annotation Pipeline (v6.6) with the Best-placed reference protein set GeneMarkS-2+ (NCBI BioProject PRJNA1036626). The resulting assembly had 240 contigs with a total length of 6,739,787 bp, 132× mean coverage, and an *N*_50_ of 49,757. DNA G + C content was 60.4 mol%, and the number of predicted proteins was 6,125. Genome completeness was estimated as 99.86% by assessing the presence of single-copy essential genes using CheckM (v1.0.18) (7). The concatenated sequences of 16S rRNA, *dnaK*, *gyrB*, *recA*, *rpoD*, and *trpB* (8) from 16 species of the *Pseudomonas gessardii* subgroup were aligned using MUSCLE aligner (9), and phylogenetic trees were prepared using Randomized Axelerated Maximum Likelihood (RAxML) (v8.2.12) (10) with 100 bootstraps and a maximum-likelihood search. This analysis showed that SED1^T^ is most closely related to *P. yamanorum* (Fig. 1). The scaffolds were analyzed by the Type Strain Genome Server (TYGS) (11), which uses Genome BLAST Distance Phylogeny and digital DNA-DNA hybridization (dDDH) values to perform species and sub-species clustering. The GGDC platform uses high-scoring segment pairs (HSPs) between related species to calculate the *d*_4_ value, which is the ratio between all identities found in HSPs and total HSP length (Table 1) (12). Pairwise comparisons with *d*_4_ < 70% are presumed to indicate that the genomes are from distinct species. The *d*_4_ value for SED1^T^ and *P. yamanorum*, the most closely related bacterium, was 58.2%, consistent with isolate SED1^T^ representing a previously undescribed species of the genus *Pseudomonas*.

**TABLE 1:** GGDC analysis. Pairwise analysis of SED1^T^ with closely related *Pseudomonas* species was performed on the TYGS (11)^*a*^

Subject strain	dDDH *d*_4_ (%)	C.I. *d*_4_ (%)	G + C difference (%)
*Pseudomonas yamanorum* LMG 27247	58.2	(55.4–60.9)	0.06
*Pseudomonas spelaei* CCM 7893	32.4	(30.0–34.9)	1.04
*Pseudomonas marginalis* DSM 13124	31.2	(28.8–33.7)	0.23
*Pseudomonas aylmerensis* S1E40	31	(28.6–33.5)	1.24
*Pseudomonas karstica* CCM 7891	30.9	(28.5–33.4)	1.51
*Pseudomonas petroselini* MAFF 311094	30.8	(28.4–33.3)	0.33
*Pseudomonas shahriarae* SWRI52	30.7	(28.3–33.2)	0.19
*Pseudomonas allii* MAFF 301514	30.7	(28.3–33.2)	0.47
*Pseudomonas marginalis* ICMP 3553	30.6	(28.2–33.1)	0.01
*Pseudomonas brenneri* DSM 15294	30.6	(28.2–33.1)	0.26
*Pseudomonas brenneri* JCM 13307	30.5	(28.1–33.0)	0.19
*Pseudomonas antarctica* LMG 22709	30.5	(28.1–33.0)	0.88
*Pseudomonas azotoformans* LMG 21611	30.4	(28.0–32.9)	0.58
*Pseudomonas extremorientalis* LMG 19695	30.4	(28.0–32.9)	0.53
*Pseudomonas synxantha* DSM 18928	29.7	(27.3–32.2)	0.74
*Pseudomonas migulae* NBRC 103157	25.8	(23.5–28.3)	1.28
*Pseudomonas piscicola* P50	25.6	(23.3–28.1)	1.9
*Pseudomonas gregormendelii* LMG 28632	25.6	(23.3–28.1)	0.79

^*a*^ *d_4_* is the ratio between all identities found in HSPs and total HSP length, and C.I. is the confidence interval.

## Data Availability

The assembly and raw data are deposited at NCBI through the SRA genome submission portal under the accession numbers JAXCIS000000000 and SRR25184390. The version deposited in this paper is the first version. The type strain is deposited in the American Type Culture Collection (ATCC) and the Culture Collection of Switzerland (CCOS) under accession numbers TSD-406 and CCOS-2108, respectively.
